# Somatic mutations and outcomes in chronic myeloid leukemia adolescent and young adults compared to children, adults, and *BCR::ABL1*-positive acute lymphoblastic leukemia

**DOI:** 10.1038/s41375-025-02609-3

**Published:** 2025-04-28

**Authors:** Jitka Krizkova, Vaclava Polivkova, Adam Laznicka, Nikola Curik, Adela Benesova, Pavla Suchankova, Tomas Smazik, Veronika Vysinova, Dana Mikulenkova, Hana Klamova, Marketa Stastna Markova, Dana Srbova, Jan Zuna, Marketa Zaliova, Jan Trka, Cyril Salek, Katerina Machova Polakova

**Affiliations:** 1 Institute of Hematology and Blood Transfusion, Prague, Czech Republic; 2 Second Faculty of Medicine, Charles University, Prague, Czech Republic; 3 Institute of Clinical and Experimental Hematology of the First Faculty of Medicine, Charles University and Institute of Hematology and Blood Transfusion, Prague, Czech Republic; 4 Institute of Pathological Physiology, First Faculty of Medicine, Charles University, Prague, Czech Republic; 5 CLIP, Department of Paediatric Haematology and Oncology, Second Faculty of Medicine, Charles University and University Hospital Motol, Prague, Czech Republic

**Keywords:** Chronic myeloid leukaemia, Acute lymphocytic leukaemia

## Abstract

Adolescent and young adults (AYAs) with chronic myeloid leukemia in chronic phase (CML-CP) reportedly respond worse to tyrosine kinase inhibitors (TKIs) than adults, potentially due to additional genetic abnormalities, including mutations in cancer-related genes (CRGs). This real-life study compared mutation profiles and their impact on outcomes in 80 AYA, 97 adult, and 16 pediatric CML-CP patients, alongside 81 *BCR::ABL1*-positive acute lymphoblastic leukemia (Ph+ ALL) patients. CRG mutations were more frequent in AYAs (25.0%) than in adults (19.6%) or children (12.5%). AYAs with Ph+ ALL exhibited higher mutational frequencies (53.3%) compared to children (26.7%) and adults (38.9%). At diagnosis, mutations in *ASXL1*, *DNMT3A*, and *TET2* dominated in CML-CP and *RUNX1*, *IKZF1*, and *BCR::ABL1* in Ph+ ALL. *ASXL1* mutations correlated with reduced progression-free survival (PFS) in AYAs and adults. Unlike adults, AYAs showed no increase in *BCR::ABL1* kinase domain mutations during TKI therapy. Nilotinib improved PFS in AYAs with *ASXL1* mutations, highlighting the efficacy of higher-generation TKIs. *ASXL1* mutations also impaired erythropoiesis, warranting further validation. Despite a higher mutational burden, AYAs did not exhibit worse prognoses than adults. Lower mutation rates at follow-up suggest potential impact of nilotinib. Mutation profiling and optimized TKI use are crucial to mitigate progression risks in CRG-mutated patients.

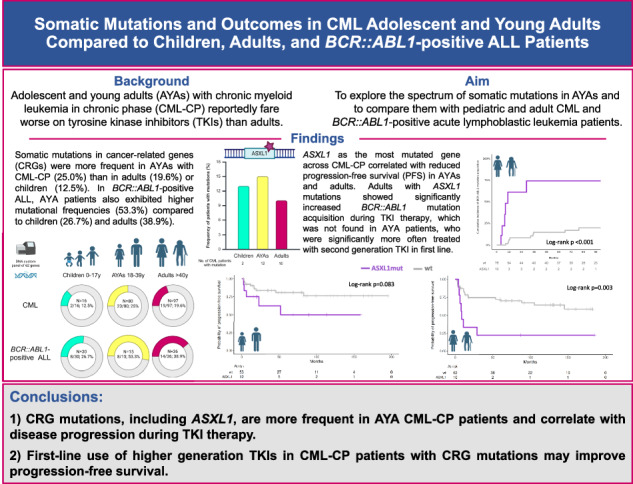

## Introduction

Chronic myeloid leukemia (CML) is characterized by a translocation t(9;22)(q34;q11.2), which results in *BCR::ABL1* rearrangement. Targeting the BCR::ABL1 protein with tyrosine kinase inhibitors (TKIs) dramatically changed outcome of CML patients. The landscape of TKI therapy has evolved with the introduction of second and third-generation TKIs that have demonstrated enhanced efficacy and a broader spectrum of *BCR::ABL1* inhibition compared to their predecessors [1, 2]. Currently, most patients with chronic phase CML (CML-CP) achieve a normal life expectancy. Factors affecting the efficiency of TKI treatment include the availability of drugs, their tolerability, the emergence of resistance to TKIs, as well as patient compliance, comorbidities, and age-related considerations [3, 4].

The incidence of CML rises with age, with the median age at diagnosis exceeding 60 years [5, 6] and the highest occurrence observed in individuals aged 75 and over. Prior to the integration of TKIs into clinical practice, advanced age was regarded as an adverse prognostic factor [7] and was involved in calculation of SOKAL and Euro scores [8, 9]. Despite adolescents and young adults (AYAs), defined by the National Comprehensive Cancer Network (NCCN) Guidelines (Version 2.2024) as patients aged 15–39, accounting for 7–10% of newly diagnosed CML cases, they remain understudied.

Several clinical trials have indicated that AYA CML patients present with elevated white blood cell counts, larger spleen sizes, and lower hemoglobin levels at diagnosis compared to their older counterparts [10, 11]. Additionally, a higher proportion of AYA patients exhibit *BCR::ABL1* transcript levels exceeding 10% on the international scale (IS) at 3 months post-TKI initiation [10]. These indicators are reflective of the potentially aggressive nature of CML in this specific age group and underscore the unique challenges faced by AYA patients in disease management. However, studies have not consistently demonstrated lower probabilities of achieving major molecular response (MMR) and complete cytogenetic response (CCgR) in AYA patients. Importantly, none of these studies have confirmed the impact of age on overall survival and progression-free survival (PFS). The lack of consistent evidence emphasizes the need for further research and comprehensive investigations of AYA CML patients.

Despite the continual improvement in CML patient outcomes, ongoing research has shown that additional genetic abnormalities, including somatic mutations in cancer-related genes (CRGs), were responsible for worse response to TKIs [12–15]. Mutations in genes encoding epigenetic modifiers such as *ASXL1*, *DNMT3A*, and *TET2* have been shown to elevate the risk of molecular relapse upon treatment discontinuation [16]. Moreover, these mutations are not isolated events but are frequently associated with clonal hematopoiesis, especially in CML patients of advanced age, typically those above 65 years [17–19]. On the other hand, patients carrying *ASXL1* mutations at the time of diagnosis are often characterized as younger individuals facing a higher risk classification [13], indicating a role of clonal evolution associated with *ASXL1* mutations in CML pathogenesis. This suggests a potential role for these mutations as prognostic markers in guiding treatment decisions and long-term management strategies for CML patients.

In this study, our objective was to explore the spectrum of somatic mutations in adolescent and young adult patients with CML in chronic phase (CML-CP) treated with adult protocol and to compare them with pediatric and adult CML patients, as well as with patients diagnosed with *BCR::ABL1*-positive acute lymphoblastic leukemia (Ph+ ALL).

## Materials and methods

### Patient cohorts

The total cohort of 193 patients diagnosed with chronic myeloid leukemia in chronic phase, classified according to European Leukemia Net (ELN) criteria [3]. Patients were treated at the Institute of Hematology and Blood Transfusion and University Hospital in Motol in Prague, Czech Republic. Detailed clinical information is given in Table 1. Despite the NCCN-recommended age definition for AYA cancer patients, we categorized patients based on treatment practices for CML and Ph+ ALL in the Czech Republic. Patients aged 0–17 years are treated in pediatric clinics following pediatric protocols, while those aged 18 and older are treated in hematology centers according to adult protocols. It is common practice that children treated under pediatric protocols transition to adult hematology centers upon reaching 18 years of age, where they continue therapy according to adult guidelines. Therefore, reflecting standard treatment practices in the Czech Republic, patients were divided into three groups based on their age at diagnosis: children (0–17 years), adolescent and young adults (AYAs) (18–39 years), and adults (>40 years). In alignment with the revised adolescent age definition proposed by Sawyer et al. [20], which considers individuals aged 10–24 years as adolescents, the AYA cohort in our study includes adolescence patients aged 18–24 years who were treated with adult protocols. The CML cohort consisted of 16 children (median 12 years; range 2–17 years), 80 AYAs (median 33 years; range 19–39 years), and 97 adults (median 58 years; range 40–79 years). Ph+ ALL cohort comprised 30 children, 15 AYAs, and 36 adult patients with median age 10 (range 2–17 years), 33 years (range 18–39 years), 56 years (range 40–77 years), respectively. All patients or their guardians provided written informed consent. The study was approved by the institutional ethical committee and performed in accordance with the Declaration of Helsinki.

**Table 1:** Baseline characteristics of AYA and adult cohorts of CML patients.

Variable	AYAs, *N* = 80^a^	Adults, *N* = 97^a^	*p*-value^b^
Age; median years (range)	33 (19, 39)	59 (40, 79)	<0.001
Sex Male	40/80 (50%)	50/97 (52%)	0.8
Spleen size, cm; median (range)	0.0 (0.0, 25.0)	0.0 (0.0, 20.0)	0.016
WBC, ×10^9^/l; median (range)	92 (9, 612)	59 (3, 500)	0.019
Platelet count, ×10^9^/l; median (range)	520 (158, 1835)	369 (86, 1778)	0.007
Hemoglobin, g/l; median (range)	119 (60, 161)	125 (14, 170)	0.033
Neutrophiles, ×10^9^/l; median (range)	98 (6, 514)	46 (2, 471)	0.026
Lymphocytes, %; median (range)	5.5 (0.0, 23.0)	7.0 (0.0, 31.0)	0.2
Monocytes, %; median (range)	2.00 (0.00, 9.00)	2.00 (0.00, 14.00)	0.051
Basophils, %; median (range)	5.0 (0.0, 24.0)	4.0 (0.0, 34.0)	0.3
Blasts, %; median (range)	1.00 (0.00, 9.00)	1.00 (0.00, 16.00)	0.078
Eosinophils, %; median (range)	2.50 (0.00, 11.00)	2.00 (0.00, 11.00)	0.2
SOKAL			0.006
Low	48/79 (61%)	37/96 (39%)	
Intermediate	17/79 (22%)	41/96 (43%)	
High	14/79 (18%)	18/96 (19%)	
EUTOS			0.2
Low	64/79 (81%)	85 / 96 (89%)	
Intermediate	0/79 (0%)	0 / 96 (0%)	
High	15/79 (19%)	11 / 96 (11%)	
ELTS			0.3
Low	61/79 (77%)	64/96 (67%)	
Intermediate	11/79 (14%)	22/96 (23%)	
High	7/79 (8.9%)	10/96 (10%)	
ACAs	3/80 (3.8%)	3/97 (3.1%)	>0.9
1st-line TKI			<0.001
Imatinib	61/80 (76%)	94/97 (97%)	
Nilotinib	19/80 (24%)	3/97 (3.1%)	

*ACAs* additional chromosomal abnormalities, *WBC* white blood count.

^a^*n*/*N* (%).

^b^Fisher’s exact test; Pearson’s Chi-squared test; Wilcoxon rank sum test.

### Primary cell isolation

Total leukocytes were isolated from peripheral blood (PB) or bone marrow (BM). Peripheral blood mononuclear cells (PMNCs) were isolated using Lymphoprep density gradient centrifugation (STEMCELL Technologies, Vancouver, Canada) according to the manufacturer´s recommendations.

### Mutation detection in *BCR::ABL1* kinase domain

*BCR::ABL1* kinase domain (*BCR::ABL1* KD) amplicon libraries were prepared using the Nextera XT DNA Library Prep Kit (Cat. No. FC-131-1096, Illumina, San Diego, CA, USA) as previously reported [21]. Data processing, error filtering, and mutation calling at significant levels were performed using the NextGENe software (Softgenetics, State College, PA, USA) and the in-house bioinformatic tool NextDom [21].

### NGS panel sequencing

DNA for NGS panel sequencing was isolated from PB/BM using MagCore (RBC Bioscience, New Taipei City, Taiwan) or from TRIzol/ITG lysates by phenol-chloroform extraction. The custom panel sequencing of 22 whole genes and the selected exons of additional 40 genes (Roche, Basel, Switzerland) frequently mutated in myeloid and lymphoid malignancies (Supplementary Table S1) was used for the detection of somatic mutations. The library was prepared using KAPA HyperPlus (Roche) according to the protocol of manufacturer and sequenced 2×150-bp on the MiSeq instrument (Illumina, San Diego, CA, USA). Data were evaluated using the NextGENe software (Softgenetics). The clinical relevance of the detected variants with minimal coverage 500× and variant allele frequencies (VAF) > 5% was evaluated using VarSome [22]. The somatic origin of mutations, where the VAF did not correspond to the level of *BCR::ABL1*, was confirmed using genomic DNA from buccal swabs.

### Response and clinical outcomes

Total RNA was isolated from PB/BM total leukocytes using standard procedures. *BCR::ABL1* transcript levels were quantified using RT-qPCR and expressed on the International Scale (IS) [23]. Response definitions and CML phases classifications followed ELN criteria [3]. The probability of progression-free survival (PFS) was estimated from the start of TKI treatment to the date of progression defined as TKI treatment failure, the presence of high-risk additional chromosomal abnormalities, *BCR::ABL1* KD mutations, or CML-related death.

### Colony forming assays

Clonogenic assays were conducted using CML progenitor CD34+ or peripheral blood mononuclear cells (PBMCs) from patients with *ASXL1* mutation detected at diagnosis (*N* = 4). Mononuclear cells were isolated using Lymphoprep separation and CD34+ cells were purified by immunomagnetic beads (CD34 MicroBead Kit Human, 130-097-047; Miltenyi Biotec, Bergish Gladbach, Germany). PBMCs were from healthy donor served as a control. CD34+ (1 × 10^3^) or PBMCs (2 × 10^5^) were seeded into methylcellulose MethoCult^TM^ H4435 medium (STEMCELL Technologies, Vancouver, Canada). Samples were analyzed in duplicate, and colonies were enumerated and characterized after 14 days. Colony counts were compared to reference progenitor cell colony frequencies in MethoCult^TM^ of healthy donors, as reported by the manufacturer.

### Statistical analysis

Baseline characteristics and hematological parameters were compared using Fisher´s exact test, Pearson´s Chi-squared or the Kruskal–Wallis tests. PFS was estimated by the Kaplan–Meier method and compared by log-rank test. Hazard ratio (HR) with a 95% confidence interval (CI) was calculated for risk factors using Cox proportional hazard regression models. The cumulative incidences of *BCR::ABL1* KD mutations were estimated using the cumulative incidence method. Univariate and multivariate analyses were performed to evaluate associations between patient characteristics and survival outcomes. Colony forming assays were evaluated using Student´s *t*-test. A statistical significance level of *p* = 0.05 was applied throughout all the experiments. All statistical analyses were performed using R 4.3.1.

## Results

### Frequency and spectrum of mutations in each age group of CML-CP and Ph+ ALL patients

To characterize the spectrum of somatic mutations in detail and to clarify the mutation landscape in AYA (*N* = 80) patients with CML-CP, we compared them with adults (*N* = 97), and pediatric patients (*N* = 16). All 193 CML patients (Table 1; Supplementary Table S2) in this study were diagnosed with chronic phase and did not progress to the blast phase during TKI treatment.

We observed that AYAs had a significantly higher white blood count (p = 0.019) and number of platelets (*p* = 0.007) compared to adults (Table 1), and even when we included children (both *p* < 0.025) (Supplementary Table S2). Furthermore, AYAs had a significantly larger spleen size compared to adult CML patients (*p* = 0.016), however, no significant difference in the percentage of blasts was observed between the two age groups (*p* = 0.078).

Among the AYAs, 76.3% (61/80) patients were treated with imatinib as the first-line therapy, while 23.8% (19/80) patients received nilotinib. In the adult group, 96.9% (94/97) patients were treated with imatinib, and 3.1% (3/97) patients were treated with nilotinib. In the pediatric cohort, all patients received imatinib as first-line treatment and 56.3% (9/16) of pediatric patients underwent hematopoietic cell transplantation. None of the patients were pretreated with interferon alpha.

**Fig. 1:**
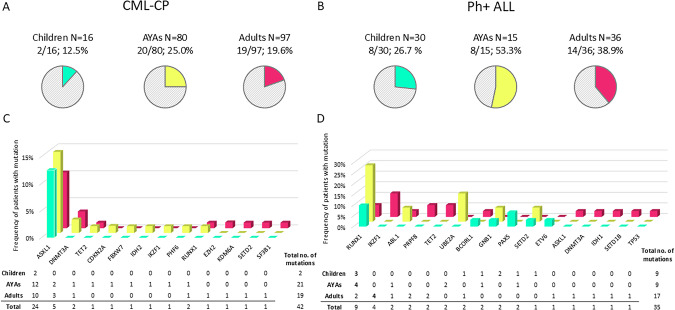
Age- and disease-related spectrum of somatic mutations at diagnosis. The frequency of patients with mutations according to age and disease (**A**) CML and (**B**) Ph+ ALL. Patients were divided into subgroups based on age at diagnosis: children (0–17 years), AYA (18–39 years), and adults (>40 years). Spectrum of somatic mutations in (**C**) CML and (**D**) Ph+ ALL patients according to age subgroups. The total number of mutations identified is shown in tables.

In total, 42 somatic mutations were identified in CML at diagnosis with a median VAF 32.2% (range 5.0–96.9) across 13 CRGs. These included 16 frameshift, 13 nonsense, 11 missense, and 2 start loss mutations (Supplementary Table S3). Somatic mutations were identified in 25% (20/80) of AYA CML patients, 19.6% (19/97) of adult patients, and 12.5% (2/16) of pediatric patients (Fig. 1A). The highest frequency of mutations was found also in Ph+ ALL AYAs (53.3%; 8/15) followed by adults (38.9%; 14/36) and children (26.7%; 8/30) (Fig. 1B).

Among the 13 CRGs mutated in CML, *ASXL1* emerged as the most frequently mutated gene in CML, with mutations observed in 2 pediatric CML patients, 13 AYA patients, and 8 adult patients (Fig. 1C). Three recurrent mutations in *ASXL1* were identified: c.1934dup G646Wfs*12, c.2077C>T R693*, and c.1773C>G Y591* affecting *N* = 8, 3, and 2 patients, respectively. All frameshift mutations led to premature stop codons with subsequent loss of the c-terminal plant-homeo-domain. Notably, only one AYA patient harbored two somatic CRG mutations. Epigenetic modifiers *ASXL1*, *DNMT3A*, and *TET2* were mutated in 18.8% (15/80) AYAs and 14.4% (14/97) of adult patients with median VAFs 31.5% (range 5.4–46.5) and 32.2% (range 5.0–48.0), respectively. No mutation in *BCR::ABL1* KD was detected in CML patients across all age groups at the time of diagnosis.

Overall, 35 somatic mutations were identified in Ph+ ALL patients with a median VAF of 44.5% (range 11.2–82.5) across 16 CRGs (Supplementary Table S3). Mutations in *RUNX1* were the most common events in both Ph+ ALL children (*N* = 3) and AYAs (*N* = 4), while mutated *IKZF1* in the adults (*N* = 4) (Fig. 1D). Mutation *RUNX1* c.602G>A R201Q was recurrently identified in two patients. Five patients were found to have two distinct somatic mutations at diagnosis. In contrast to CML patients with no *BCR::ABL1* KD mutation at diagnosis, two Ph+ ALL patients harbored *BCR::ABL1* KD mutations at the time of diagnosis.

### Frequency of CML patients with mutations in relation to TKI response

We next analyzed 177 paired samples from 80 AYA and 97 adult CML patients collected during TKI treatment, based on sample availability. At the time of follow-up sample analysis, patients were divided into TKI responders and non-responders according to ELN criteria [3] and analyzed for CRG mutations; AYA optimal responders median time 14 months since TKI start (6–87 months), warning signs median 36 months (10–164 months), therapy failure median 36 months (5–177 months); adult optimal responders median time 15 months since TKI start (6–31 months), warning median 13 months (12–52 months), failure median 24 months (5–167 months). Additionally, all the paired samples from the time of non-optimal TKI response (warning and failure) were analyzed for *BCR::ABL1* KD mutations.

Overall, the frequency of CML patients with CRG mutations, regardless of TKI response and age, was 22% (39/177) at diagnosis and 25.4% (45/177) at TKI follow-up (Supplementary Table S4). Contrary to samples at the time of diagnosis, a significantly higher prevalence of CRG mutations was found in adults 32.0% (31/97) compared to AYA patients 17.5% (14/80) in TKI follow-up (*p* = 0.04). All the detected mutations are listed in Supplementary Table S3. Adult patients developed significantly more de novo mutations (both in *BCR::ABL1* KD and other CRG) during treatment (27.8%) compared to AYA patients (12.5%) (*p* = 0.02).

At diagnosis, CRG mutations in optimal responders were slightly more frequently observed in AYAs (17.1%; 7/41) than in adult patients (12.5%; 6/48; *p* = 0.56). All 13 mutations identified in responders from both age groups except those in *EZH2* c.2T>C M1T and *DNMT3A* c.1609T>C C537R, disappeared during the TKI treatment or were observed at low VAF corresponding to the residual level of *BCR::ABL1* transcript. De novo mutations, namely *ASXL1* c.1934dup G646Wfs*12, *TET2* c.2429del Q810Rfs*3, and *DNMT3A* c.1591G>A D531N, were found only in adult responders (Supplementary Table S3).

**Fig. 2:**
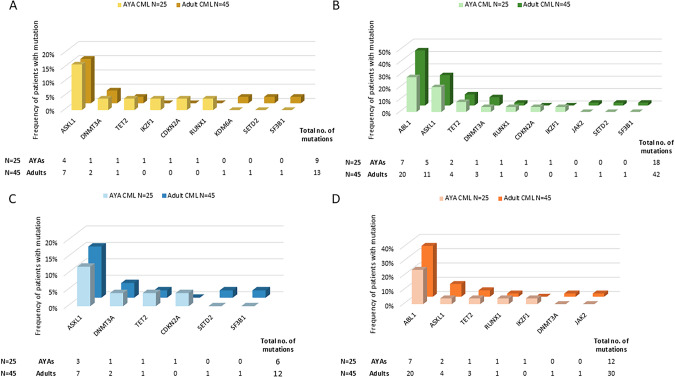
Spectrum of somatic mutations observed in AYA and adult TKI non-responders. The bars represent the frequency of patients with somatic mutations at diagnosis (**A**), TKI follow-up (**B**), mutations persisting during TKI treatment (**C**), and those acquired de novo during TKI treatment (**D**). The tables below indicate the total number of mutations detected.

In diagnostic samples of TKI non-responders, somatic mutations were detected in 9 different CRGs (Fig. 2A). All 7 *ASXL1* mutations detected in adults at diagnosis and 3/4 in AYAs persisted during TKI treatment and were detectable at TKI failure (Fig. 2B, C). While mutations in *ASXL1* most often appeared at diagnosis, mutations in *BCR::ABL1* KD were the most common genetic alterations acquired during the therapy (Fig. 2D). The frequency of de novo *BCR::ABL1* KD mutations was higher in adult patients (35.6%) compared to AYAs (24.0%). The treatment failure was also associated with the occurrence of de novo mutations in *ASXL1*, *TET2*, and *RUNX1* in both age groups. Most failures in AYA and adult patients were at the time of follow-up sample analysis treated with imatinib 1st-line (Supplementary Table S5).

### Impact of somatic mutations on outcomes of CML-CP patients

**Fig. 3:**
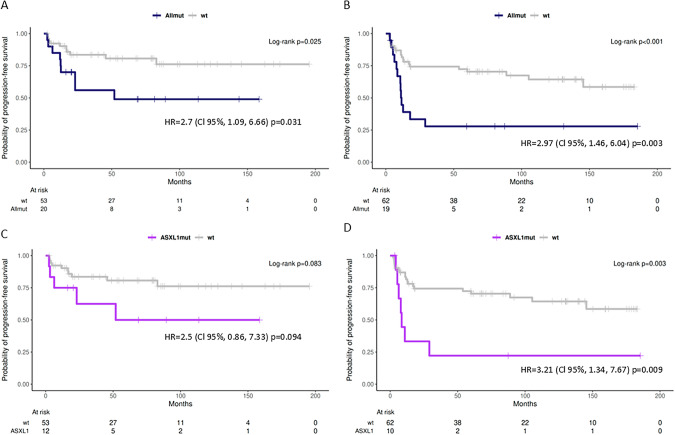
Impact of somatic mutations identified at diagnosis on PFS according to type of mutations and age groups. PFS of (**A**) AYA and (**B**) adult CML patients with any mutation identified at diagnosis compared to patients with no mutation. Effect of *ASXL1* mutations observed at diagnosis on PFS in (**C**) AYA and (**D**) adult patients. Hazard R (95% CI) derived from Cox proportional hazard regression models and the *p*-value calculated by the Log-rank test are shown. The number of patients at risk is shown in tables below. Allmut—patients with any mutation at diagnosis; wt - patients with no mutation during the whole TKI follow-up.

We evaluated the impact of somatic mutations detected in CRGs at diagnosis (20/80 AYA and 19/97 adult patients) and TKI follow-up (14/80 AYA and 31/97 adult patients) on progression-free survival (PFS) of CML patients. The presence of any mutation at diagnosis significantly reduced the probability of PFS compared to patients with no mutation both in AYA (*p* = 0.031; HR = 2.7; CI 1.09–6.66) and adult (*p* = 0.003; HR = 2.97; CI 1.46–6.04) CML patients (Fig. 3A, B). *ASXL1* mutations identified at diagnosis were associated with inferior PFS compared to patients with no mutation in both age groups, AYA (*p* = 0.094; HR = 2.5; CI 0.86–7.33) and adult (*p* = 0.009; HR = 3.21; CI 1.34–7.67) patients (Fig. 3C, D). The most common reason of therapy failure in AYAs was *BCR::ABL1* transcript level >1% at any time after 12 months of TKI treatment (8/80; 10%) and in adult patients the *BCR::ABL1* KD mutation acquisition (12/97; 12%).

Univariate analysis further revealed that high prognostic scores (SOKAL, EUTOS, ELTS) were predictive of poor outcomes in adult patients but not in AYA patients (Table 2). ELTS and EUTOS scores were highly significant in the adults *p* < 0.001; HR = 7; CI 3.29–14.89 and *p* = 0.001; HR = 3.4, CI 1.66–6.98, respectively. In AYAs, the treatment with nilotinib significantly reduced the risk of progression (*p* = 0.05; HR = 0.23; CI 0.05–0.98). De novo CRG mutations significantly worsened the PFS in AYA (*p* = 0.002; HR = 6.1, CI 1.98*–*18.75), while they showed a trend toward increased risk in adult patients, though not statistically significant (*p* = 0.07; HR = 2.04, CI 0.95–4.36).

**Table 2:** Univariate analysis of factors predicting PFS in AYA and adult CML patients.

	AYAs			Adults		
Variable	HR	95% CI	*p*-value	HR	95% CI	*p*-value
High SOKAL	1.67	0.67-4.18	0.28	1.91	1.01-3.63	**0.05**
High EUTOS	1.94	0.77-4.89	0.16	3.4	1.66-6.98	**0.001**
High ELTS	0.84	0.2-3.56	0.81	7	3.29-14.89	**<0.001**
Nilotinib	0.23	0.05-0.98	**0.05**	2.12	0.51-8.79	0.30
*ASXL1* at diagnosis	2.5	0.86-7.33	0.09	3.21	1.34-7.67	**0.009**
Any mutation at diagnosis	2.7	1.09-6.66	**0.03**	2.97	1.46-6.04	**0.003**
De novo non*BCR::ABL1* mutation	6.1	1.98-18.75	**0.002**	2.04	0.95-4.36	0.07

The bold values mean that the analysed factor for PFS prediction was statistically significant.

Univariate Cox Regression.

### Impact of *ASXL1* mutations on cumulative incidence of *BCR::ABL1* kinase domain mutations during follow-up on TKI treatment

**Fig. 4:**
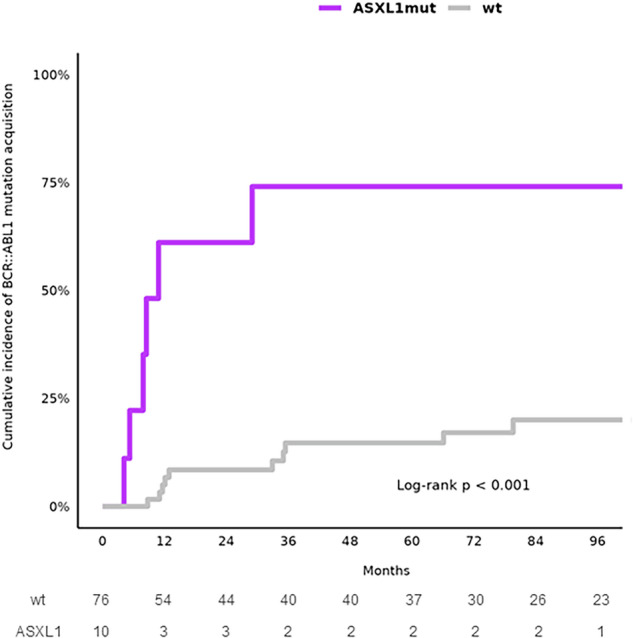
Cumulative incidence of *BCR::ABL1* KD mutations in adult CML patients according to the presence of *ASXL1* mutation at the time of diagnosis. The number of patients at risk is shown on the table below. ASXL1mut *ASXL1* mutation at diagnosis, wt no mutation at diagnosis.

Cumulative incidence of mutations in *BCR::ABL1* KD acquired during TKI treatment was significantly higher in adult patients with *ASXL1* mutation at the time of diagnosis compared to adult patients with no mutation at diagnosis (*p* < 0.001) (Fig. 4). Five of six adult patients with *ASXL1* mutation at diagnosis that acquired *BCR::ABL1* KD mutation were treated with imatinib. Contrary to adults, only one AYA patient (Patient #13) with nonsense mutation *ASXL1* E773* at diagnosis developed de novo mutations in *BCR::ABL1* KD (F317L and M351T) (Supplementary Table S3).

### Impact of *ASXL1* mutations on clonogenicity

Next, we assessed the clonogenic potential of CD34+ cells and PBMCs from three patients with *ASXL1* mutations, from whom the cells were available and vital (Supplementary Table S6). Blood count results showed reduced or minimal erythropoiesis at the time of diagnosis and anemia or mild anemia in Pt 2 and Pt 3 (Supplementary Table S7). Impaired erythropoiesis was observed in CD34+ cells, evidenced by a decrease in CFU-GM (Pt 1) of erythroid progenitor colonies (Supplementary Fig. S1, Panel A). In patients 2 and 3, PBMCs were available only for the clonogenicity analysis, which is not ideal sample as isolated CD34 + . However, impaired erythropoiesis was noted in PBMCs from a patient with the *ASXL1* E877 frameshift mutation (Pt 3), as indicated by a decrease in BFU-E (*p* < 0.001), CFU-E (*p* < 0.001), and CFU-GEMM numbers (*p* < 0.01) (Supplementary Fig. S1, Panel B).

Additionally, we analyzed the blood counts of all AYA patients (*N* = 11) and all adults (*N* = 10) with the *ASXL1* mutation (Supplementary Table S8). For comparison, we randomly selected 28 AYAs and 28 adults from the studied cohorts without *ASXL1* mutations and evaluated their blood counts. Patients with the *ASXL1* mutation (*n* = 20) exhibited a significantly higher platelet count compared to those without the mutation (*N* = 56) (*p* < 0.001). This difference remained statistically significant when analyzed within age groups (AYA patients with *ASXL1* mutation vs. non-mutated *ASXL1*: *p* = 0.02; adults with *ASXL1* mutation vs. non-mutated *ASXL1*: *p* = 0.016). As mentioned above, a higher platelet count was observed in AYA patients compared to adults. Moreover, an even higher platelet level was noted in AYA patients with *ASXL1* mutations.

## Discussion

This work focused on AYA CML-CP patients, whose outcome on TKI therapy has been previously reported as worse compared to adult patients [24]. The CML-CP cohort consisted of 80 AYAs, 97 adults and 16 pediatric patients. At diagnosis, AYAs exhibited significantly larger spleen sizes and higher levels of white blood cells and platelets compared to adult patients. This is consistent with studies reporting that younger CML patients often present more risk factors compared to older patients [10, 11]. There were also differences in baseline characteristics and treatment regimens; a greater proportion of AYA patients received the second-generation TKI nilotinib as a first-line treatment compared to adult patients, aligning with the observations of Castagnetti et al. [11], who noted age-based variations in TKI usage.

Altogether, 42 somatic mutations in 13 CRGs were identified, with a higher mutation frequency in AYA CML patients (25.0%) compared to adults (19.6%) and pediatric (12.5%) patients treated in real-clinical practice. Additionally, among Ph+ ALL patients, AYA individuals were diagnosed with CRG mutations more frequently (53.3%) than children (26.7%) and adult patients (38.9%). The elevated mutational burden in AYAs in both diseases is notable. The landscape of mutated genes at diagnosis differed between CML-CP and Ph+ ALL patients. Among 193 CML-CP patients, the most frequently mutated genes were *ASXL1*, *DNMT3A*, and *TET2*, which is consistent with recent studies [14, 15, 25]. In contrast, the most frequently mutated genes in 81 Ph+ ALL patients were *RUNX1*, *IKZF1*, and *BCR::ABL1* KD. However, Feng et al. [26]. reported these mutations in Ph+ ALL patients but with lower frequency compared to mutations in genes involved in transcriptional regulation and epigenetic modulation, namely *FAT1*, *CRLF2*, *SF1*, *EP300*, and *CREBBP* (all these genes are included in our panel except for *FAT1*).

*ASXL1* was the most frequently mutated gene at diagnosis across pediatric (2/16; 13%), AYA (12/80; 15%), and adult (10/97; 10%) CML patients. Our findings align with those of Ernst et al. [19], who reported a higher prevalence of *ASXL1* mutations in young patients (29%) compared to adult CML-CP patients (7–13%). Similarly, *ASXL1* was the most frequently mutated gene in pediatric CML patients (6/90; 6.7%) [27]. Notably, *ASXL1* mutations were significantly associated with an increased cumulative incidence of *BCR::ABL1* KD mutations during TKI therapy in adult CML patients, whereas this association was not observed in AYAs. Likewise, Guerineau et al. [28] demonstrated on a cohort of CML patients that was not divided into age groups that the presence of mutations in epigenetic genes at diagnosis was linked to a higher cumulative incidence of *BCR::ABL1* KD mutations (*p* = 0.015). These findings suggest that mutations in epigenetic modulators may promote additional genetic events, such as *BCR::ABL1* KD mutations, contributing to therapeutic failure. CML patients with significantly lower probability of PFS were those who carried mutated *ASXL1* in both AYAs and adult patients in comparison with patients without mutations supporting the assumption that *ASXL1* is the CML-related oncogene [13]. Furthermore, *ASXL1* mutations may contribute to impaired erythropoiesis, as suggested by blood count abnormalities and reduced colony formation from erythroid progenitors, which is in line with findings from previous studies [29, 30]. Although several studies [10, 11, 31] have reported the elevated platelet counts in younger CML patients, very little is known about the association of increased platelet counts and mutations in *ASXL1*. Behrens et al. [27] has reported that pediatric CML patients harboring pathogenic *ASXL1* mutations at diagnosis exhibited higher counts of leukocytes and platelets compared to patients without any mutation or other mutation than *ASXL1*, which may be a potential consequence of the relative increase in proliferative capacity in the study cohort. The elevated level of platelets in patients with *ASXL1* mutation compared to patients without mutation has also been reported in adult CML patients, but not statistically significant [32].

Although CRG mutations were detected at diagnosis of CML at a lower frequency, their presence correlated with reduced PFS and enhanced the level of significance. Together, these findings support emerging evidence that mutations in CRGs detected at the time of diagnosis in CML-CP patients represent risk factors for disease progression. Based on *BCR::ABL1* transcript kinetics and variant allele frequency (VAF) of mutations in CRGs, it is presumed that these mutations are present in CML cells, which is in line with previous works [19, 25].

*BCR::ABL1* was the most frequent gene with mutation acquisition during TKI therapy with a markedly higher prevalence in adult patients, suggesting age-related susceptibility to additional mutations under TKI treatment pressure. This pattern aligns with Kim et al. [25], who reported higher rates of new mutations in older CML patients during TKI therapy. *ASXL1* was the second most frequently mutated gene on TKI therapy, albeit at a much lower frequency than *BCR::ABL1*.

Univariate analysis revealed that high SOKAL, EUTOS and ELTS scores along with *ASXL1* mutations were significantly associated with reduced PFS in adult patients. This pattern was not observed in AYAs, where nilotinib therapy showed significant association with PFS. Specifically, nilotinib was administered to 50% (6/12) of AYA patients with *ASXL1* mutations, highlighting that more potent TKIs than imatinib, when used as first-line therapy in patients with *ASXL1* mutations, may improve PFS.

As this study is based on real-world data, this hypothesis requires validation in larger cohorts of patients treated with higher-generation TKIs as a first-line approach. Conversely, previous work based on clinical trial data indicated that patients with *ASXL1* mutations had inferior probability to achieve MMR on nilotinib as first-line therapy [13]. However, this study did not compare outcomes in patients treated with imatinib to evaluate the MMR rates in patients with *ASXL1* mutations.

We acknowledge certain limitations of our study. First, the cohort of pediatric CML patients is relatively small, as CML is rare in this population. Consequently, statistical conclusions cannot be drawn from this subgroup alone. However, the inclusion of pediatric patients was primarily intended to provide a comprehensive overview of mutation spectra across all age groups for comparative purposes. Our analyses focused predominantly on AYA and adult patients, particularly those who were not initially referred for alloHSCT. Despite the low prevalence of CML in children and the rarity of somatic mutations in CRGs at diagnosis, we successfully characterized the spectrum of somatic mutations across all age categories.

Second, while our findings suggest that first-line treatment with second- or third-generation TKIs may lead to better outcomes in patients carrying *ASXL1* mutations compared to those treated with imatinib as a first-line therapy, further validation through clinical studies is required. Additionally, the observed impact of *ASXL1* mutations on impaired erythropoiesis should be investigated in a larger cohort, specifically comparing patients with *ASXL1* mutations to those without CRG mutations.

In conclusion, this comparative study on mutation frequency and mutational landscapes in AYA, pediatric, and adult patients with CML-CP and Ph+ ALL revealed that CRG mutations were more frequently detected in AYA patients at the time of diagnosis. However, our findings did not support the initial hypothesis that AYA CML-CP patients might carry oncogenic mutations commonly observed in Ph+ ALL. This study demonstrated that mutations in CRGs in CML-CP patients (both AYA and adults) represent a risk factor for disease progression during TKI therapy. In general, CML-CP patients who responded optimally to TKI therapy exhibited lower mutation rates at both diagnosis and follow-up, particularly among AYA patients treated with nilotinib. Patients who did not respond to TKI therapy (treatment failures) exhibited higher mutation rates both at diagnosis and during follow-up. Nevertheless, adult patients generally showed higher mutation rates at follow-up, irrespective of response, suggesting a potential age-related factor. *ASXL1* mutations and other CRG mutations serve as risk factors for progression during TKI therapy in both AYA and adult CML-CP patients. Although overall, AYA do not seem to have a worse prognosis than others, despite having more mutations. Using higher generations of TKIs at diagnosis that effectively target CML cells with *ASXL1* mutations and possibly other CRGs could potentially reduce disease progression risk.

## Supplementary information

Supplementary Information

## Data Availability

The datasets generated during and/or analyzed during the current study are available from the corresponding author on reasonable request.
