# Supplementary material for: Somatic mutations and outcomes in chronic myeloid leukemia adolescent and young adults compared to children, adults, and BCR::ABL1-positive acute lymphoblastic leukemia
Source: Leukemia. 2025 Apr 28;39(7):1670–7. doi: 10.1038/s41375-025-02609-3 (PMC12208900; doi:10.1038/s41375-025-02609-3)
Supplement: Supplementary file 1 — Supplementary Information [file 41375_2025_2609_MOESM1_ESM.pdf]

Supplementary Information

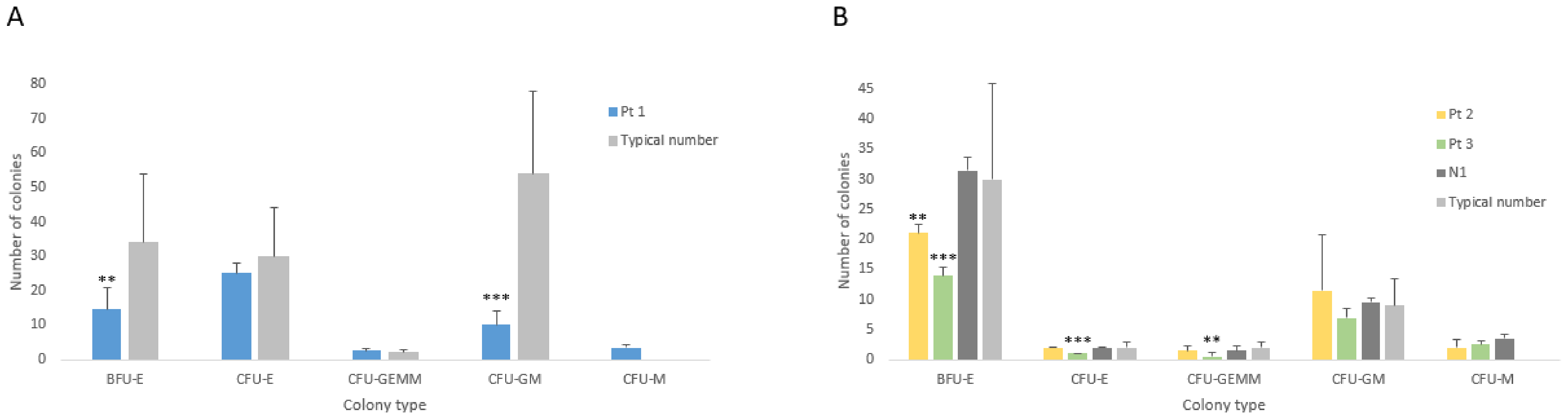

Supplementary Figure S1. **Impact of *ASXL1* mutations on clonogenicity of hematopoietic progenitors.** Number of colonies originated from (A) CD34+ and (B) PBMCs after growing 14 days in semi-solid medium. BFU-E – Burst forming unit – erythroid; CFU-E – Colony forming unit – Erythroid; CFU-GEMM – Colony forming unit - Common myeloid progenitor; CFU-GM – Colony forming unit - Granulocyte–macrophage progenitor; CFU-M – Colony forming unit – Macrophage. Error bars represent standard deviation. ** $p<0.01$; *** $p<0.001$

Supplementary Table S1. Full and partly covered genes in custom NGS panel.

Gene			Gene	Exons
BCOR	full		ABL1	4-10
BCORL1	full		ASXL1	12
CDKN2A	full		ATRX	8-10, 17-31
CEBPA	full		BCL2	2 ,3 both partly
DNMT3A	full		BRAF	15
ETV6	full		CBL	8, 9
EZH1	full		CBLB	9, 10
EZH2	full		CREBBP	24-30
GNB1	full		CRLF2	6
GNB2	full		CSF3R	14-17
IKZF1	full		EP300	24-30
KDM6A	full		FBXW7	9-11
PAX5	full		FLT3	14, 15, 20
PHF6	full		GATA1	2
PRPF8	full		GATA2	2-6
RUNX1	full		IDH1	4
SETD1B	full		IDH2	2, 4
SETD2	full		JAK1	14-16
SF1	full		JAK2	12, 14
SIRT1	full		JAK3	13
UBE2A	full		KIT	2, 8-19
ZRSR2	full		KRAS	2, 3
**total gene full**		**22**	MCL1	1, 2, 3 partly
			MLL (KMT2A)	5-11
			MPL	10
			NF1	18
			NOTCH1	26-28, 34
			NPM1	11
			NRAS	2, 3
			PDGFRA	12, 14, 18
			PTEN	5, 7
			PTPN11	3, 13
			SETBP1	4 partly
			SF3B1	12-16
			SMC3	10,13,19,23,25+28
			SRSF2	1
			TET2	3-11
			TP53	2-11
			U2AF1	2, 6
			WT1	4-9
			**total gene partly**	**40**

Supplementary Table S2. Baseline characteristics of CML children.

Variable	N	N = 16	p-value[1]
Age; median (range)	16	12.0 (2.0, 17.0)	
Sex Male	16	11 / 16 (69%)	
Spleen size, cm; median (range)	9	4.0 (0.0, 14.0)	
WBC, x 10^9/l; median (range)	15	123 (37, 406)	0.025
Platelet count, x 10^9/l; median (range)	14	448 (158, 1,285)	0.025
Hemoglobin, g/l; median (range)	14	113 (70, 148)	0.035
Lymphocytes, %; median (range)	15	4.0 (1.0, 24.0)	
Monocytes, %; median (range)	14	3.65 (0.00, 7.00)	
Blasts, %; median (range)	13	0.90 (0.00, 7.00)	
Eosinophils, %; median (range)	14	2.5 (0.5, 12.0)	
Allo_SCT	16	9 / 16 (56%)	

[1]P-value was calculated compared to AYA and adult CML patients using Pearson's Chi-squared test

Supplementary Table S3 A. Detected mutations in CML patients at diagnosis.

Age group	Patient #	HGVS	Chr	Position hg38	Type	VAF (%)
Children	#3	ASXL1(NM_015338.6):c.2077C>T p.(Arg693Ter)	20	32434789	Nonsense	10.1
Children	#12	ASXL1(NM_015338.6):c.1934dup p.(Gly646TrpfsTer12)	20	32434639	Frameshift	33.2
AYA_optimal	#1	IDH2(NM_002168.4):c.419G>A p.(Arg140Gln)	15	90088702	Missense	28.9
AYA_optimal	#2	ASXL1(NM_015338.6):c.2077C>T p.(Arg693Ter)	20	32434789	Nonsense	5.4
AYA_optimal	#3	DNMT3A(NM_022552.5):c.1792C>T p.(Arg598Ter)	2	25467083	Nonsense	39.1
AYA_optimal	#4	ASXL1(NM_015338.6):c.1934dup p.(Gly646TrpfsTer12)	20	32434639	Frameshift	9.6
AYA_optimal	#5	ASXL1(NM_015338.6):c.2329G>T p.(Glu777Ter)	20	32435041	Nonsense	9.2
AYA_optimal	#6	PHF6(NM_001015877.2):c.1024C>T p.(Arg342Ter)	X	134425256	Nonsense	49.1
AYA_optimal	#7	ASXL1(NM_015338.6):c.1773C>G p.(Tyr591Ter)	20	32434485	Nonsense	8.5
AYA_warning	#1	ASXL1(NM_015338.6):c.4118_4119del p.(Phe1373CysfsTer6)	20	32436828	Frameshift	36.5
AYA_warning	#2	ASXL1(NM_015338.6):c.2077C>T p.(Arg693Ter)	20	32434789	Nonsense	25.8
AYA_warning	#3	ASXL1(NM_015338.6):c.2491_2492dup p.(Asp832LeufsTer7)	20	32435201	Frameshift	36.3
AYA_warning	#4	FBXW7(ENST00000281708.10):c.1513C>T p.(Arg505Cys)	4	152326137	Missense	20.7
AYA_warning	#4	ASXL1(NM_015338.6):c.3111G>A p.(Trp1037Ter)	20	32435823	Missense	35.2
AYA_failure	#1	ASXL1(NM_015338.6):c.2383_2384insG p.(Ser795CysfsTer5)	20	32435096	Frameshift	10.7
AYA_failure	#2	IKZF1(NM_006060.6):c.550C>T p.(Arg184Trp)	7	50382668	Missense	10.9
AYA_failure	#3	ASXL1(NM_015338.6):c.1934dup p.(Gly646TrpfsTer12)	20	32434639	Frameshift	39.1
AYA_failure	#4	ASXL1(NM_015338.6):c.2634_2647del p.(Ser878ArgfsTer11)	20	32435343	Frameshift	31.5
AYA_failure	#5	CDKN2A(NM_058197.5):c.2T>A p.(Met1Lys)	9	21974826	Start loss	48.0
AYA_failure	#12	RUNX1(ENST00000675419.1):c.494G>A p.(Gly165Asp)	21	34880571	Missense	5.5
AYA_failure	#13	ASXL1(NM_015338.6):c.2317G>T p.(Glu773Ter)	20	32435029	Nonsense	46.5
AYA_failure	#14	TET2(NM_001127208.3):c.5602C>G p.(His1868Asp)	4	105276112	Missense	8.9
AYA_failure	#15	DNMT3A(NM_022552.5):c.1904G>A p.(Arg635Gln)	2	25243930	Missense	45.1
Adult_optimal	#1	ASXL1(NM_015338.6):c.1921_1929delATCGGAGGGinsGT p.(Ile641ValfsTer60)	20	32434633	Frameshift	23.5
Adult_optimal	#2	ASXL1(NM_015338.6):c.1934dup p.(Gly646TrpfsTer12)	20	32434639	Frameshift	27.6
Adult_optimal	#3	ASXL1(NM_015338.6):c.1934dup p.(Gly646TrpfsTer12)	20	32434639	Frameshift	32.0
Adult_optimal	#4	RUNX1(ENST00000675419.1):c.480C>A p.(Asp160Glu)	21	34880585	Missense	22.6
Adult_optimal	#6	EZH2(ENST00000320356.7):c.2T>C p.(Met1Thr)	7	148847297	Start loss	42.0
Adult_optimal	#47	DNMT3A(NM_022552.5):c.1609T>C p.(Cys537Arg)	2	25244598	Missense	47.0
Adult_failure	#1	DNMT3A(NM_022552.5):c.2313del p.(Leu773SerfsTer6)	2	25463180	Frameshift	41.9
Adult_failure	#2	ASXL1(NM_015338.6):c.2929C>T p.(Gln977Ter)	20	31023444	Nonsense	34.3
Adult_failure	#3	SF3B1(NM_012433.4):c.1867T>C p.(Tyr623His)	2	197402766	Missense	42.3
Adult_failure	#4	DNMT3A(ENST00000321117.10):c.2617C>T p.(His873Tyr)	2	25234401	Missense	5.0
Adult_failure	#5	KDM6A(NM_001291415.2):c.1553C>G p.(Ser518Ter)	X	45061391	Nonsense	96.9
Adult_failure	#6	TET2(NM_001127208.3):c.2298del p.(Asn766LysfsTer47)	4	105236240	Frameshift	48.0
Adult_failure	#7	ASXL1(NM_015338.6):c.1934dup p.(Gly646TrpfsTer12)	20	32434639	Frameshift	25.3
Adult_failure	#8	ASXL1(NM_015338.6):c.1900_1922del p.(Glu635ArgfsTer15)	20	32434600	Frameshift	32.3
Adult_failure	#9	ASXL1(NM_015338.6):c.1934dup p.(Gly646TrpfsTer12)	20	32434639	Frameshift	8.8
Adult_failure	#10	ASXL1(NM_015338.6):c.2388G>A p.(Trp796Ter)	20	32435100	Nonsense	5.0
Adult_failure	#11	ASXL1(NM_015338.6):c.1773C>G p.(Tyr591Ter)	20	32434485	Nonsense	46.6
Adult_failure	#12	ASXL1(NM_015338.6):c.1934dup p.(Gly646TrpfsTer12)	20	32434639	Frameshift	32.7
Adult_failure	#13	SETD2(NM_014159.7):c.4027C>T p.(Gln1343Ter)	3	47120609	Nonsense	41.1

Supplementary Table S3 B. Detected mutations in CML patients at follow up.

Age group	Patient #	HGVS	Chr	Position hg38	Type	VAF (%)	De novo
AYA_optimal	#3	DNMT3A(NM_022552.5):c.1792C>T p.(Arg598Ter)	2	25467083	Nonsense	5.0	
AYA_warning	#5	ASXL1(NM_015338.6):c.1934del p.(Gly645ValfsTer58)	20	32434639	Frameshift	6.1	De novo
AYA_failure	#3	ASXL1(NM_015338.6):c.1934dup p.(Gly646TrpfsTer12)	20	32434639	Frameshift	36.8	
AYA_failure	#4	ASXL1(NM_015338.6):c.2634_2647del p.(Ser878ArgfsTer11)	20	32435343	Frameshift	27.6	
AYA_failure	#5	CDKN2A(NM_058197.5):c.2T>A p.(Met1Lys)	9	21974826	Start loss	52.4	
AYA_failure	#6	ASXL1(NM_015338.6):c.1934dup p.(Gly646TrpfsTer12)	20	32434639	Frameshift	22.8	De novo
AYA_failure	#7	ABL1(NM_005157.6):c.730A>G p.(Met244Val)	9	130862943	Missense	1.2	De novo
AYA_failure	#8	ABL1(NM_005157.6):c.1135G>A p.(Val379Ile)	9	130874917	Missense	0.2	De novo
AYA_failure	#9	ABL1(NM_005157.6):c.1250C>A p.(Ser417Tyr)	9	130875032	Missense	0.3	De novo
AYA_failure	#10	ABL1(NM_005157.6):c.730A>G p.(Met244Val)	9	130862943	Missense	35.6	De novo
AYA_failure	#11	ABL1(NM_005157.6):c.749G>A p.(Gly250Glu)	9	130862962	Missense	5.0	De novo
AYA_failure	#13	ASXL1(NM_015338.6):c.2317G>T p.(Glu773Ter)	20	32435029	Nonsense	44.2	
AYA_failure	#13	ABL1(NM_005157.6):c.951C>G p.(Phe317Leu)	9	130872903	Missense	9.3	De novo
AYA_failure	#13	ABL1(NM_005157.6):c.1052T>C p.(Met351Thr)	9	130873004	Missense	30.4	De novo
AYA_failure	#14	TET2(NM_001127208.3):c.5602C>G p.(His1868Asp)	4	105276112	Missense	10.0	
AYA_failure	#14	RUNX1(NM_001754.5):c.1268_1273delGCTCGCinsT p.(Arg423LeufsTer175)	21	34792305	Frameshift	27.0	De novo
AYA_failure	#14	TET2(NM_001127208.3):c.2435del p.(Ile812LysfsTer12)	4	105236377	Frameshift	13.0	De novo
AYA_failure	#15	DNMT3A(NM_022552.5):c.1904G>A p.(Arg635Gln)	2	25243930	Missense	36.2	
AYA_failure	#15	IKZF1(NM_006060.6):c.484C>T p.(Arg162Trp)	7	50382602	Missense	35.5	De novo
AYA_failure	#15	ASXL1(NM_015338.6):c.1934dup p.(Gly646TrpfsTer12)	20	32434639	Frameshift	25.9	De novo
Adult_optimal	#5	ASXL1(NM_015338.6):c.1934dup p.(Gly646TrpfsTer12)	20	32434639	Frameshift	23.1	De novo
Adult_optimal	#6	EZH2(ENST00000320356.7):c.2T>C p.(Met1Thr)	7	148847297	Start loss	48.8	
Adult_optimal	#6	TET2(NM_017628.4):c.2429del p.(Gln810ArgfsTer3)	4	105236371	Frameshift	5.5	De novo
Adult_optimal	#47	DNMT3A(NM_022552.5):c.1609T>C p.(Cys537Arg)	2	25244598	Missense	11.0	
Adult_optimal	#47	DNMT3A(ENST00000321117.10):c.1591G>A p.(Asp531Asn)	2	25244616	Missense	6.0	De novo
Adult_failure	#1	DNMT3A(NM_022552.5):c.2313del p.(Leu773SerfsTer6)	2	25463180	Frameshift	5.0	
Adult_failure	#2	ASXL1(NM_015338.6):c.2929C>T p.(Gln977Ter)	20	31023444	Nonsense	5.1	
Adult_failure	#3	SF3B1(NM_012433.4):c.1867T>C p.(Tyr623His)	2	197402766	Missense	36.1	
Adult_failure	#4	DNMT3A(ENST00000321117.10):c.2617C>T p.(His873Tyr)	2	25234401	Missense	12.1	
Adult_failure	#5	TET2(NM_001127208.3):c.1576C>T p.(Gln526Ter)	4	105235518	Nonsense	6.9	De novo
Adult_failure	#6	TET2(NM_001127208.3):c.2298del p.(Asn766LysfsTer47)	4	105236240	Frameshift	35.1	
Adult_failure	#6	TET2(NM_001127208.3):c.3100C>T p.(Gln1034Ter)	4	105237042	Nonsense	10.9	De novo
Adult_failure	#6	TET2(NM_001127208.3):c.3845G>A p.(Gly1282Asp)	4	105259660	Missense	5.9	De novo
Adult_failure	#7	ASXL1(NM_015338.6):c.1934dup p.(Gly646TrpfsTer12)	20	32434639	Frameshift	10.3	
Adult_failure	#7	ABL1(NM_005157.6):c.749G>A p.(Gly250Glu)	9	130862962	Missense	4.5	De novo
Adult_failure	#7	ABL1(NM_005157.6):c.1052T>C p.(Met351Thr)	9	130873004	Missense	7.7	De novo
Adult_failure	#8	ASXL1(NM_015338.6):c.1900_1922del p.(Glu635ArgfsTer15)	20	32434600	Frameshift	9.1	
Adult_failure	#8	ABL1(NM_005157.6):c.749G>A p.(Gly250Glu)	9	130862962	Missense	5.0	De novo
Adult_failure	#8	ABL1(NM_005157.6):c.1052T>C p.(Met351Thr)	9	130873004	Missense	9.8	De novo
Adult_failure	#9	ASXL1(NM_015338.6):c.1934dup p.(Gly646TrpfsTer12)	20	32434639	Frameshift	36.5	
Adult_failure	#9	ABL1(NM_005157.6):c.951C>G p.(Phe317Leu)	9	130872903	Missense	46.2	De novo
Adult_failure	#10	ASXL1(NM_015338.6):c.2388G>A p.(Trp796Ter)	20	32435100	Nonsense	9.5	
Adult_failure	#10	ABL1(NM_005157.6):c.763G>A p.(Glu255Lys)	9	130862976	Missense	15.5	De novo
Adult_failure	#11	ASXL1(NM_015338.6):c.1773C>G p.(Tyr591Ter)	20	32434485	Nonsense	26.6	
Adult_failure	#11	ABL1(NM_005157.6):c.764A>T p.(Glu255Val)	9	130862977	Missense	5.6	De novo
Adult_failure	#12	ASXL1(NM_015338.6):c.1934dup p.(Gly646TrpfsTer12)	20	32434639	Frameshift	5.2	
Adult_failure	#12	ABL1(NM_005157.6):c.635C>A p.(Thr212Lys)	9	130862848	Missense	5.1	De novo
Adult_failure	#13	SETD2(NM_014159.7):c.4027C>T p.(Gln1343Ter)	3	47120609	Nonsense	5.0	

Adult_failure	#13	ABL1(NM_005157.6):c.730A>G p.(Met244Val)	9	130862943	Missense	5.1	De novo
Adult_failure	#14	ABL1(NM_005157.6):c.764A>T p.(Glu255Val)	9	130862977	Missense	5.0	De novo
Adult_failure	#15	ABL1(NM_005157.6):c.756G>T p.(Gln252His)	9	130862969	Missense	33.7	De novo
Adult_failure	#16	ABL1(NM_005157.6):c.896T>C p.(Val299Ala)	9	130872202	Missense	5.0	De novo
Adult_failure	#17	ABL1(NM_005157.6):c.749G>A p.(Gly250Glu)	9	130862962	Missense	5.4	De novo
Adult_failure	#17	ABL1(NM_005157.6):c.757T>C p.(Tyr253His)	9	130862970	Missense	5.3	De novo
Adult_failure	#18	ABL1(NM_005157.6):c.933C>G p.(Phe311Leu)	9	130872885	Missense	5.1	De novo
Adult_failure	#19	ABL1(NM_005157.6):c.730A>G p.(Met244Val)	9	130862943	Missense	5.0	De novo
Adult_failure	#20	ABL1(NM_005157.6):c.944C>T p.(Thr315Ile)	9	130872896	Missense	37.0	De novo
Adult_failure	#21	ABL1(NM_005157.6):c.1358A>T p.(Glu453Val)	9	130878502	Missense	5.0	De novo
Adult_failure	#22	ABL1(NM_005157.6):c.1159T>A p.(Leu387Met)	9	130874941	Missense	38.8	De novo
Adult_failure	#23	ASXL1(NM_015338.6):c.2077C>T p.(Arg693Ter)	20	32434789	Nonsense	11.1	
Adult_failure	#23	ABL1(NM_005157.6):c.951C>G p.(Phe317Leu)	9	130872903	Missense	5.4	De novo
Adult_failure	#24	RUNX1(ENST00000675419.1):c.1003del p.(Gln335SerfsTer259)	21	34792576	Frameshift	6.7	De novo
Adult_failure	#24	DNMT3A(NM_022552.5):c.2074C>T p.(Gln692Ter)	2	25241570	Nonsense	6.0	De novo
Adult_failure	#25	ASXL1(NM_015338.6):c.1934dup p.(Gly646TrpfsTer12)	20	32434639	Frameshift	16.1	De novo
Adult_failure	#26	ASXL1(NM_015338.6):c.1934dup p.(Gly646TrpfsTer12)	20	32434639	Frameshift	19.1	De novo
Adult_failure	#27	JAK2(ENST00000381652.4):c.1849G>T p.(Val617Phe)	9	5073770	Missense	5.0	De novo
Adult_failure	#28	ASXL1(NM_015338.6):c.2324T>G p.(Leu775Ter)	20	32435036	Nonsense	14.0	De novo

Supplementary Table S3 C. Detected mutations in Ph+ ALL patients at diagnosis.

Age group	Patient #	HGVS	Chr	Position hg38	Type	VAF (%)
Children	#3	ETV6(NM_001987.5):c.195_202delCGTAGCCCinsGG p.(Asp65_Gln68delinsGluGlu)	12	11839171	In frame	37.2
Children	#3	BCORL1(ENST00000540052.6):c.3586C>T p.(Arg1196Ter)	X	130021129	Nonsense	45.1
Children	#6	RUNX1(ENST00000675419.1):c.611G>A p.(Arg204Gln)	21	34859476	Missense	45.7
Children	#10	RUNX1(ENST00000675419.1):c.958C>T p.(Arg320Ter)	21	34799310	Nonsense	44.7
Children	#13	GNB1(NM_001282539.2):c.275C>A p.(Ala92Asp)	12	1804574	Missense	16.8
Children	#16	PAX5(ENST00000358127.9):c.154T>G p.(Cys52Gly)	9	37020694	Missense	30.9
Children	#26	RUNX1(NM_001754.5):c.319C>T p.(Arg107Cys)	21	34886875	Missense	49.0
Children	#27	PAX5(NM_001280552.2):c.630_631insAGCCCCC p.(Gly211SerfsTer34)	9	36966699	Frameshift	34.7
Children	#32	SETD2(NM_014159.7):c.6528_6531delTGTGinsGGGA p.(Tyr2176Ter)	3	47057253	Nonsense	35.4
AYA	#2	SETD2(NM_014159.7):c.7514_7516dup p.(Phe2505_Lys2506insIle)	3	47017655	In frame	60.9
AYA	#3	RUNX1(NM_001754.5):c.1090_1103del p.(Ile364ValfsTer231)	21	34792475	Frameshift	35.7
AYA	#3	ABL1(NM_005157.6):c.730A>G p.(Met244Val)	9	130862943	Missense	45.5
AYA	#4	UBE2A(NM_003336.4):c.263G>A p.(Cys88Tyr)	X	119582609	Missense	11.2
AYA	#5	GNB1(NM_002074.5):c.265A>G p.(Lys89Glu)	1	1806477	Missense	14.3
AYA	#6	RUNX1(NM_001754.5):c.601C>T p.(Arg201Ter)	21	34859486	Nonsense	46.4
AYA	#7	RUNX1(NM_001754.5):c.602G>A p.(Arg201Gln)	21	34859485	Missense	46.5
AYA	#10	RUNX1(NM_001754.5):c.400G>C p.(Ala134Pro)	21	34880665	Missense	36.9
AYA	#11	UBE2A(NM_003336.4):c.262T>C p.(Cys88Arg)	X	119582608	Missense	81.7
Adult	#1	TET2(NM_001127208.3):c.3332T>A p.(Leu1111Ter)	4	105237274	Nonsense	41.5
Adult	#1	RUNX1(NM_001754.5):c.502G>A p.(Gly168Arg)	21	34880563	Missense	45.1
Adult	#2	ASXL1(NM_015338.6):c.2077C>T p.(Arg693Ter)	20	32434789	Nonsense	40.0
Adult	#3	ABL1(NM_005157.6):c.944C>T p.(Thr315Ile)	9	130872896	Missense	*
Adult	#6	SETD1B(NM_001353345.2):c.844C>A p.(Pro282Thr)	12	121809789	Missense	29.5
Adult	#11	TP53(NM_000546.6):c.821T>C p.(Val274Ala)	17	7673799	Missense	80.9
Adult	#14	IKZF1(NM_006060.6):c.575T>C p.(Leu192Pro)	7	50382693	Missense	45.4
Adult	#15	IKZF1(NM_006060.6):c.919A>T p.(Lys307Ter)	7	50399986	Nonsense	41.7
Adult	#21	RUNX1(ENST00000675419.1):c.602G>A p.(Arg201Gln)	21	34859485	Missense	44.5
Adult	#21	BCORL1(NM_021946.5):c.1251_1253delGAGinsTCCCTCGGCTGGAA p.(Leu417PhefsTer31)	X	130014023	Frameshift	14.2
Adult	#24	TET2(NM_001127208.3):c.5618T>C p.(Ile1873Thr)	4	105276128	Missense	44.5
Adult	#26	IKZF1(NM_006060.6):c.173_177del p.(Lys58ArgfsTer5)	7	50376543	Frameshift	82.5
Adult	#28.	IDH1(NM_005896.4):c.394C>T p.(Arg132Cys)	2	208248389	Missense	38.4
Adult	#29	PRPF8(ENST00000703541.1):c.211C>T p.(Arg71Ter)	17	1683591	Nonsense	26.8
Adult	#33	IKZF1(NM_006060.6):c.460T>C p.(Phe154Leu)	7	50382578	Missense	62.7
Adult	#36	DNMT3A(NM_175629.2):c.852C>A p.(Tyr284Ter)	2	25248040	Nonsense	62.7
Adult	#36	PRPF8(ENST00000572621.5):c.4780T>C p.(Cys1594Arg)	17	1660437	Missense	48.9

* Mutation was detected by ddASO assay for T315I with positive result

Supplementary Table S4. The frequency of CML patients with CRG mutations at diagnosis and follow up.

No. of pts with mutation (%)	Regardless TKI response			Optimal (TKI responders)			Failure (TKI non-responders)			Warning		
	AYAs N=80	Adults N=97	p-value	AYAs N=41	Adults N=48	p-value	AYAs N=25	Adults N=45	p-value	AYAs N=14	Adults N=4	p-value
at diagnosis	20 (25.0%)	19 (19.6%)	0.47	7 (17.1%)	6 (12.5%)	0.56	9 (36.0%)	13 (28.9%)	0.60	4 (28.6%)	0 (0%)	0.52
at TKI follow up	14 (17.5%)	31 (32.0%)	**0.04***	1 (2.4%)	3 (6.3%)	0.62	12 (48.0%)	28 (62.2%)	0.32	1 (7.1%)	0 (0%)	1.0
persisting from dg	7 (8.8%)	14 (14.4%)	0.35	1 (2.4%)	2 (4.2%)	1.0	6 (24.0%)	12 (26.7%)	1.0	0 (0%)	0 (0%)	1.0
de novo any mutation	10 (12.5%)	27 (27.8%)	**0.02***	0 (0%)	3 (6.3%)	0.25	9 (36.0%)	24 (53.3%)	0.21	1 (7.1%)	0 (0%)	1.0
de novo BCR::ABL1 mutation	6 (7.5%)	16 (16.5%)	0.11	0 (0%)	0 (0%)	1.0	6 (24.0%)	16 (35.6%)	0.42	0 (0%)	0 (0%)	1.0
de novo non*BCR::ABL1* mutation	4 (5.0%)	11 (11.3%)	0.18	0 (0%)	3 (6.3%)	0.25	3 (12.0%)	8 (17.8%)	0.73	1 (7.1%)	0 (0%)	1.0

pts - patients; Fisher´s exact test * $p<0.05$

Supplementary Table S5. The TKI treatment of CML patients during the follow up.

		AYAs (N=80)			Adults (N=97)		
**Line of treatment**	**TKI**	**Optimal**	**Warning**	**Failure**	**Optimal**	**Warning**	**Failure**
1st	IM/NILO/DASA	23/13/0	8/5/0	19/1/0	44/1/0	4/0/0	32/1/0
2nd	IM/NILO/DASA	0/0/4	-	0/0/3	0/2/1	-	0/3/3
3rd	IM/NILO/DASA	0/0/1	0/0/1	0/1/0	-	-	0/1/0
4th	IM/NILO/DASA/BOSU/PONA	-	-	0/1/0/0/0	-	-	0/0/1/1/1
other/no TKI		-	-	-	-	-	2

Supplementary Table S6. Characteristics of patients with *ASXL1* mutation at diagnosis tested on clonogenicity of CD34+ and PBMCs cells.

	Diagnosis					Time of sample collection					
**Sample**	**Phase of disease at Dg**	**Type of material**	***BCR::ABL1* % IS**	**ASXL1 mutation (VAF %)**	**Other mutations (VAF %)**	**Phase of disease**	**TKI therapy (number of line)**	***BCR::ABL1* % IS**	**ASXL1 mutation (VAF %)**	**Other mutations (VAF %)**	**Other mutations (VAF %)**
Pt 1	CML-CP	CD34+	137	W1037X (35%)	FBXW7 R505C (21%)	CML-CP	no	137	W1037X (35%)	FBXW7 R505C (21%)	no
Pt 2	CML-CP	PBMCs	26	Y591X (47%)	no	CML-CP	ASCI (3rd)	3.6	Y591X (3%)	ABL1 M244V (81%)	ABL1 E255V (1%)
Pt 3	CML-CP	PBMCs	110	E877delinsRfs (21%)	no	CML-CP	ASCI (4th)	1.8	E877delinsRfs (1%)	no	no
N1	Healthy donor	PBMCs	-	-	-	-	-	-	-	-	-

Pt - patient; N - healthy donor; PBMCs - peripheral blood mononuclear cells; ASCI - asciminib

Supplementary Table S7. The blood counts of patients tested on clonogenicity.

		Pt 1	Pt 2		Pt 3	
	References	Dg=analysis	Dg	Analysis	Dg	Analysis
Erythrocytes, 10^12/L	4.00-5.80	4.22	3.6	3.82	*ND*	4.51
Hemoglobin, g/L	135-175	130	106	134	71	138
Hematocrit, L/L	0.400-0.500	0.397	0.297	0.383	*ND*	0.393
Mean Corpuscular Volume (MCV), fL	82.0-98.0	94.1	82.5	100.3	71.9	87.1
Mean Corpuscular Hemoglobin (MCH), pg	28.0-34.0	30.8	29.4	35.1	*ND*	30.6
Mean Corpuscular Hemoglobin Concentration (MCHC), g/L	320-360	327	357	350	*ND*	351
Red Cell Distribution Width (RDW), %	10.00-15.20	14.2	17.7	17.7	*ND*	14.5
Platelets, 10^9/L	150-400	983	277	82	1079	54
Mean Platelet Volume (MPV), fL	7.8-12.8	10.7	11.7	13	*ND*	*ND*
Platelet Distribution Width (PDW), fL	9.0-17.0	12.3	14.5	23.5	*ND*	*ND*
Plateletcrit (PCT), mL/L	1.20-3.50	10	3	1.1	*ND*	*ND*
Erythroblasts, 10^9/L	0.00-0.00	0	1.42	0	*ND*	0
Erythroblasts, /100WBC	0.00-0.00	0	0.6	0	*ND*	0
Reticulocytes absolute, 10^12/L	0.025-0.100	0.07	0.067	0.083	*ND*	0.076
Reticulocytes relative, %	0.50-2.50	1.66	1.87	2.17	*ND*	1.69
Mean Reticulocyte Hemoglobin Content, pg	28.0-35.0	33.2	26.8	36.1	*ND*	32.6
Immature Reticulocyte Fraction (IRF), %	2.4-17.5	21.4	42.8	32.5	*ND*	14.8

Dg - at diagnosis; Analysis - at the time of analysis; *ND* - not determined

Supplementary Table S8. The blood counts of patients with or without ASXL1 mutation at diagnosis.

	Regardless age			AYAs			Adults		
	**ASXL1**	**nonASXL1**		**ASXL1**	**nonASXL1**		**ASXL1**	**nonASXL1**	
**Characteristic - Median (Min, Max)**	N = 21	N = 56	**p-value**[1]	N = 11	N = 28	**p-value**[1]	N = 10	N = 28	**p-value**[1]
Erythrocytes	3.92 (2.81, 5.18)	4.07 (1.89, 5.74)	0.9	3.74 (2.81, 5.18)	3.81 (1.89, 5.74)	0.4	4.07 (3.37, 5.08)	4.14 (2.15, 5.58)	0.5
Hemoglobin	118 (84, 148)	122 (60, 170)	>0.9	119 (84, 148)	112 (60, 154)	0.3	117 (92, 143)	128 (64, 170)	0.2
Hematocrit	0.36 (0.25, 0.46)	0.37 (0.18, 0.50)	>0.9	0.36 (0.25, 0.46)	0.34 (0.18, 0.45)	0.3	0.36 (0.30, 0.43)	0.39 (0.20, 0.50)	0.14
Mean Corpuscular Volume (MCV)	88.2 (80.0, 99.2)	89.8 (78.3, 108.3)	0.7	88.5 (80.0, 99.2)	87.2 (78.3, 97.4)	0.4	87.4 (81.6, 94.5)	90.9 (79.1, 108.3)	0.2
Mean Corpuscular Hemoglobin (MCH)	29.50 (26.30, 32.70)	29.80 (23.70, 36.90)	0.3	29.80 (26.30, 32.70)	29.90 (23.70, 32.70)	0.7	29.10 (27.30, 30.90)	29.75 (26.20, 36.90)	0.2
Immature Reticulocyte Fraction (IRF)	26 (16, 43)	26 (9, 52)	0.9	24 (16, 31)	26 (9, 52)	0.3	26 (19, 43)	26 (10, 44)	0.5
Mean Corpuscular Hemoglobin Concentration (MCHC)	329 (311, 357)	334 (299, 401)	0.3	329 (320, 341)	337 (299, 401)	0.11	329 (311, 357)	330 (308, 372)	0.8
Red Cell Distribution Width (RDW)	16.60 (14.20, 26.60)	16.80 (0.23, 29.20)	0.7	15.90 (14.20, 21.30)	17.45 (0.23, 29.20)	0.3	17.65 (15.60, 26.60)	16.25 (13.30, 20.60)	0.054
Platelet	796 (229, 1,778)	450 (66, 1,605)	**<0.001**	836 (488, 1,358)	535 (158, 1,605)	**0.020**	698 (229, 1,778)	360 (66, 1,174)	**0.016**
Mean Platelet Volume (MPV)	10.70 (7.00, 13.40)	10.10 (7.00, 12.60)	0.12	10.60 (7.00, 12.00)	9.90 (7.00, 12.50)	0.072	10.85 (7.10, 13.40)	10.45 (7.00, 12.60)	0.6
Platelet Distribution Width (PDW)	13 (9, 74)	12 (0, 74)	0.2	13 (12, 74)	12 (0, 74)	0.2	13 (9, 64)	13 (10, 67)	0.6
Plateletcrit (PCT)	1.47 (0.20, 15.90)	1.02 (0.13, 12.70)	0.059	1.44 (0.38, 10.10)	1.33 (0.15, 10.40)	0.2	2.2 (0.2, 15.9)	0.8 (0.1, 12.7)	0.2
Immature Platelet Fraction (IPF)	5.35 (1.60, 13.80)	3.50 (1.00, 13.80)	0.071	5.90 (1.60, 9.70)	3.20 (1.60, 6.90)	0.13	4.8 (2.6, 13.8)	4.6 (1.0, 13.8)	0.3
Erythroblasts litre	0.18 (0.00, 2.23)	0.20 (0.00, 14.44)	0.7	0.21 (0.00, 2.23)	0.25 (0.00, 6.83)	0.8	0.13 (0.00, 1.42)	0.20 (0.00, 14.44)	0.7
Erythroblasts_100WBC	0.35 (0.00, 0.80)	0.20 (0.00, 4.40)	0.5	0.40 (0.00, 0.80)	0.20 (0.00, 3.00)	0.5	0.30 (0.00, 0.60)	0.30 (0.00, 4.40)	0.8

[1] Fisher's exact test; Wilcoxon rank sum test
